# Ultrasonographic and Clinical Outcomes of Pulsed Ultrasound Versus Pulsed Shortwave Diathermy in Mild-to-Moderate Carpal Tunnel Syndrome: A Randomized Controlled Trial

**DOI:** 10.3390/healthcare14162555

**Published:** 2026-08-15

**Authors:** Zeynel Ertürk, Bilgehan Kolutek Ay

**Affiliations:** Department of Physical Medicine and Rehabilitation, Faculty of Medicine, Kahramanmaras Sutcu Imam University, 46040 Kahramanmaras, Türkiye

**Keywords:** carpal tunnel syndrome, ultrasonography, diathermy, median nerve, randomized controlled trial

## Abstract

**Background:** To compare the clinical and ultrasonographic effects of pulsed therapeutic ultrasound (US) and pulsed shortwave diathermy (SWD) in patients with mild-to-moderate carpal tunnel syndrome (CTS). **Methods:** In this randomized controlled trial, 40 patients with electrophysiologically confirmed mild-to-moderate CTS were randomly allocated into two groups. Both groups received wrist splinting and tendon–nerve gliding exercises. The US group received 15 sessions of pulsed ultrasound therapy (3 MHz, 0.8 W/cm^2^), whereas the SWD group received 15 sessions of pulsed shortwave diathermy (80 Hz, 19.2 W). Outcomes were assessed at baseline, 1 month, and 3 months using ultrasonographic median nerve cross-sectional area (MNCSA) (primary outcome), Boston Carpal Tunnel Questionnaire (BCTQ), Visual Analog Scale (VAS) for pain, Douleur Neuropathique 4 (DN4), and handgrip strength. **Results:** Baseline demographic characteristics, including age (mean ± SD) and sex distribution, were comparable between groups (*p* > 0.05), although BCTQ Functional Status and Total scores differed significantly at baseline. Repeated-measures analyses demonstrated significant time effects for MNCSA and all secondary clinical outcomes, including BCTQ scores, VAS pain, DN4 scores, and grip strength (all *p* < 0.001; partial η^2^ range: 0.51–0.75), indicating moderate-to-large effect sizes. Both the US and SWD groups showed sustained improvements at the 1- and 3-month follow-ups, with no significant differences between treatment modalities (all group × time interaction *p* > 0.05). **Conclusions:** In patients with mild-to-moderate CTS, both US and SWD administered alongside splinting and exercise were associated with significant clinical and ultrasonographic improvements over 3 months. No significant difference was observed between the two modalities. Both treatments may be considered adjunctive options within conservative management, although further controlled studies are needed to clarify modality-specific effects.

## 1. Introduction

Carpal tunnel syndrome (CTS) is a clinical condition caused by compression of the median nerve within the carpal tunnel at the wrist and is considered the most common entrapment neuropathy of the upper extremity. Clinically, it presents with paresthesia, pain, nocturnal numbness, and, in advanced cases, reduced grip strength, significantly affecting activities of daily living [1,2].

Current clinical practice guidelines recommend conservative management as the first-line treatment for patients with mild-to-moderate CTS, whereas surgical decompression is generally reserved for those with severe CTS, evidence of median nerve denervation, or persistent symptoms despite appropriate conservative treatment [3].

Conservative management includes wrist splinting, exercise therapy, corticosteroid injections, and physical therapy modalities, with treatment selection based on symptom severity, patient characteristics, and the available evidence [4,5]. Among conservative treatment options, wrist splinting and nerve-gliding exercises are commonly recommended as standard interventions, whereas physical therapy modalities may be used as adjunctive treatments to improve pain and function [3,4,5].

Therapeutic ultrasound (US), particularly when applied in pulsed mode, may exert non-thermal biological effects. Experimental evidence suggests that it may enhance cell membrane permeability, improve local microcirculation, and modulate inflammatory activity. These effects may help reduce intraneural edema, improve the microenvironment surrounding the compressed median nerve, and facilitate recovery of nerve function in CTS. Clinical studies have reported that ultrasound therapy can reduce symptom severity and functional limitations in patients with CTS [1,2,4].

Shortwave diathermy (SWD), on the other hand, may induce biophysical responses in deep tissues through the delivery of electromagnetic energy. In pulsed applications, non-thermal effects are predominant and may contribute to clinical improvement by enhancing local blood flow and promoting tissue metabolism. Randomized controlled trials have demonstrated that pulsed SWD may improve symptom severity, functional status, and certain electrophysiological measures in patients with CTS [6].

Although both pulsed US and pulsed SWD are widely used in the conservative management of CTS, randomized controlled trials directly comparing these modalities remain scarce. Most previous studies have primarily evaluated clinical symptoms and electrophysiological outcomes, whereas evidence regarding ultrasonography-assessed changes in median nerve morphology remains limited [5,6,7]. Therapeutic US is commonly used as part of conservative management for CTS [4,5], whereas pulsed SWD represents an alternative physical therapy modality that may be particularly useful when direct contact is not feasible, local intolerance is present, or a different biophysical mechanism is desired [6,7]. Because these modalities differ in their mechanisms of action and practical application, determining their relative effectiveness may facilitate evidence-based treatment selection and optimize conservative management strategies for CTS. Consequently, the comparative effects of pulsed US and pulsed SWD on both clinical and ultrasonographic outcomes remain unclear. Therefore, this study aimed to compare the clinical and ultrasonographic effects of pulsed US and pulsed SWD, administered in addition to standard conservative treatment consisting of wrist splinting and nerve-gliding exercises, in patients with electrophysiologically confirmed mild-to-moderate CTS.

## 2. Methods

### 2.1. Study Design, Ethical Approval, and Registration

This study was designed as a parallel-group, assessor-blinded, randomized controlled trial. The trial was designed and reported in accordance with the Consolidated Standards of Reporting Trials (CONSORT) guidelines [8]. The trial was retrospectively registered at ClinicalTrials.gov (NCT07545161) on 16 April 2026.

### 2.2. Sample Size Calculation and Participants

Sample size calculation was performed using G*Power software (version 3.1) for a repeated-measures ANOVA assessing the group × time interaction. A large effect size (f = 0.40) was selected based on the magnitude of treatment effects reported in previous randomized controlled trials evaluating conservative physical therapy interventions in patients with mild-to-moderate CTS [9,10]. With an alpha level of 0.05, a statistical power of 80%, two groups, and three repeated measurements, the minimum required total sample size was calculated to be 28 participants. To improve the reliability and precision of the analyses and to account for potential participant loss during follow-up, 40 participants were enrolled.

A total of 61 patients who presented to the outpatient clinic of Physical Medicine and Rehabilitation were screened for eligibility. Forty patients who met the inclusion and exclusion criteria were enrolled in the study.

The inclusion criteria were as follows: age between 18 and 65 years, symptom duration of at least four weeks, and a diagnosis of mild-to-moderate carpal tunnel syndrome confirmed by electromyography according to the Padua neurophysiological classification [9,11].

Patients were excluded if they had cervical radiculopathy, brachial plexopathy, thoracic outlet syndrome, previous carpal tunnel surgery, severe carpal tunnel syndrome, pregnancy, malignancy, coagulation disorders, cardiac pacemaker, or a history of physical therapy for carpal tunnel syndrome within the previous six months [2,6,7]. In participants with bilateral CTS, only the dominant hand was included in the statistical analyses to ensure the independence of observations. To minimize potential confounding effects, participants were instructed not to use analgesic medications or receive any other treatment for CTS during the week preceding enrollment and throughout the study period.

**Figure 1:**
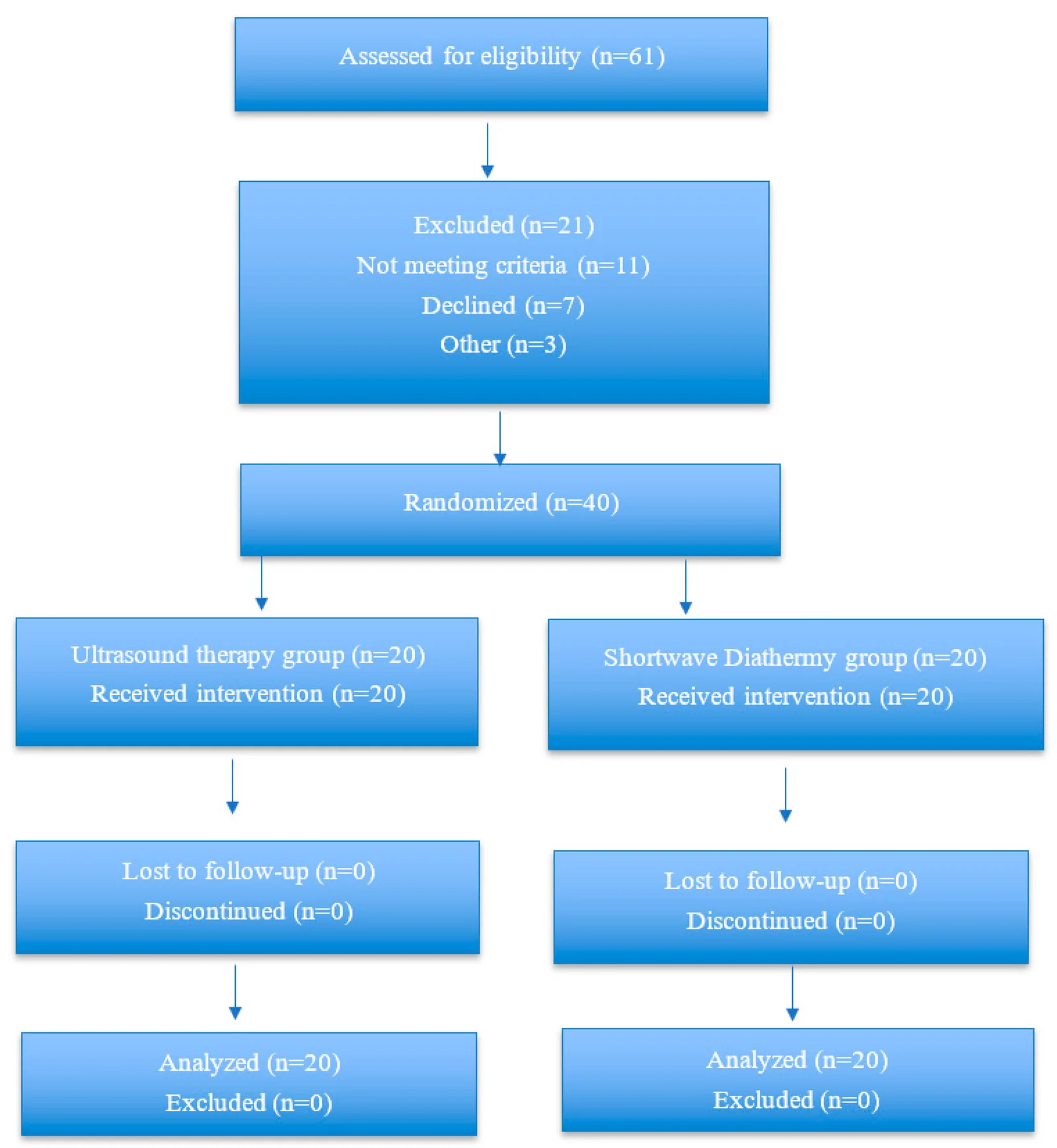
CONSORT flow diagram of participant recruitment and follow-up.

The participant flow diagram is presented in Figure 1.

### 2.3. Randomization and Blinding

Eligible participants were randomly assigned in a 1:1 ratio to either the pulsed US group or the pulsed SWD group. A simple randomization sequence was generated using a computer-based random number generator by an independent researcher who was not involved in participant recruitment, baseline assessment, treatment administration, or outcome assessment. Neither block randomization nor stratified randomization was used.

To ensure allocation concealment, group assignments were recorded in a coded allocation list that was inaccessible to the investigators responsible for participant recruitment and baseline assessment. The allocation for each participant was disclosed only to the physiotherapist responsible for administering the intervention after completion of all baseline assessments.

Due to the nature of the interventions, neither the participants nor the treating physiotherapists could be blinded to treatment allocation. However, all outcome assessments were performed by researchers who were blinded to group allocation and were not involved in treatment administration. Ultrasonographic measurements of the median nerve cross-sectional area and handgrip strength were performed by a blinded researcher. Clinical outcomes, including the Boston Carpal Tunnel Questionnaire, the Visual Analog Scale for pain, and the Douleur Neuropathique 4 questionnaire, were assessed face-to-face by another independent blinded researcher.

Outcome assessors had no access to the randomization sequence, allocation list, or treatment records. Participants were instructed not to disclose their treatment allocation to the outcome assessors during the follow-up assessments. All assessment data were recorded using participant identification codes. The success of assessor blinding was not formally evaluated.

### 2.4. Interventions

Both groups received a total of 15 treatment sessions over a three-week period. All participants were instructed to use a neutral-position wrist splint for at least four hours per night and were taught median nerve and tendon gliding exercises, performed as three sets of ten repetitions daily [12,13].

In the US group, US was applied using a 2023 model Chattanooga Intelect Neo^®^ device (Chattanooga, TN, USA). A 2 cm^2^ treatment head was positioned over the volar aspect of the wrist, centered at the distal palmar crease. The treatment parameters were set at a frequency of 3 MHz, an intensity of 0.8 W/cm^2^, a pulsed ratio of 1:4, and a duration of 5 min per session [13,14]. The ultrasound intensity remained constant at 0.8 W/cm^2^ throughout the treatment period for all participants. No adjustments were required during the intervention, as the treatment was well tolerated by all participants.

In the SWD group, a 2013 model Chattanooga Intelect Shortwave 400^®^ device (Chattanooga, Germany) was used. Electrodes were placed parallel to the volar and dorsal surfaces of the wrist, centered at the distal palmar crease. The treatment was delivered in pulsed mode with a frequency of 80 Hz, a mean output power of 19.2 W, and a duration of 15 min per session [6,7].

Adverse events were systematically assessed at each treatment session and follow-up visit. Specifically, participants were monitored for increased pain, skin erythema, burns, local swelling, excessive heat sensation, allergic reactions to the ultrasound gel, and worsening of neurological symptoms. No intervention-related adverse events or harms were observed during the study period.

### 2.5. Outcome Measures

The primary outcome of the study was the change in ultrasonographically measured MNCSA from baseline to the third month. Secondary outcomes included the total and subscale scores of the BCTQ, VAS, neuropathic pain assessed using the DN4, handgrip strength, and changes in MNCSA at other assessment time points. All evaluations were performed at baseline, at one month, and at three months.

*Ultrasonographic Assessment:* The transverse MNCSA (mm^2^) was measured using a 2024 model Acuson NX3 Elite ultrasound system (Siemens Healthineers^®^, Seoul, Republic of Korea) equipped with a 5–16 MHz high-frequency linear-array transducer. Participants were examined in the supine position with the shoulder slightly abducted, the elbow extended, the forearm supinated, and the wrist maintained in a neutral position. Measurements were performed at the level of the distal palmar crease, between the pisiform and scaphoid bones, using a manual tracing technique along the inner border of the hyperechoic epineurial rim [15]. All measurements were conducted by a specialist physician with at least five years of clinical and ultrasonography experience. Three measurements were obtained for each participant, and the mean value was used for statistical analysis.

*VAS*: Pain and paresthesia severity were assessed using a 10 cm horizontal VAS, where 0 indicated “no pain” and 10 indicated “the worst imaginable pain.” The VAS is a valid and reliable method for pain assessment [16].

*BCTQ*: The BCTQ was developed by Levine et al. [17] and consists of two subscales assessing symptom severity and functional status. Each item is scored on a 5-point Likert scale, and mean scores are calculated for each subscale. Higher scores indicate worse clinical status. The Turkish validity and reliability of the questionnaire were established by Sezgin et al. [18].

*DN4*: The DN4 questionnaire was developed by Bouhassira et al. [19]. It consists of 10 items, and a total score of ≥4 indicates the presence of neuropathic pain. The Turkish validity and reliability study was conducted by Çevik et al. [20]. In the present study, DN4 was analyzed both as a continuous variable and categorically according to the ≥4 cut-off value.

*Handgrip Strength*: Handgrip strength was measured using a Jamar^®^ hydraulic hand dynamometer in accordance with the standardized position recommended by the American Society of Hand Therapists. Participants were seated upright with the shoulder adducted and neutrally rotated, the elbow flexed at 90°, the forearm in neutral position, and the wrist in slight extension. Three measurements were obtained, and the mean value in kilograms (kg) was used for analysis [21].

### 2.6. Statistical Analysis

All statistical analyses were performed using IBM SPSS Statistics version 22 (IBM Corp., Armonk, NY, USA). Continuous variables were presented as mean ± standard deviation, and categorical variables were expressed as frequencies and percentages. The normality of continuous variables was assessed using the Shapiro–Wilk test.

To evaluate changes in the primary outcome (MNCSA), a two-way repeated measures analysis of variance (ANOVA) was performed with group (two levels) and time (three measurement points) as factors. The same model was applied to secondary continuous outcome measures. The main effects of time, group, and the group × time interaction were examined separately. The assumption of sphericity was tested using Mauchly’s test, and the Greenhouse–Geisser correction was applied when this assumption was violated. When a significant main effect of time was observed, pairwise post hoc comparisons were performed using the Bonferroni correction. Effect sizes were reported as partial eta squared (η^2^p). DN4 scores were analyzed both as a continuous variable within the two-way repeated measures ANOVA model and as a categorical variable using the ≥4 cut-off value. For categorical DN4 data, within-group changes over time were evaluated using Cochran’s Q test, and between-group comparisons at each time point were performed using Fisher’s exact test. Baseline continuous variables were compared using the independent-samples *t*-test, whereas categorical variables were compared using the Pearson χ^2^ test or Fisher’s exact test, as appropriate. Between-group comparisons at the 1- and 3-month follow-up assessments were performed using the independent-samples *t*-test, and mean differences with 95% confidence intervals (CIs) were reported. Because baseline differences were observed in the Boston Functional Status Score and Boston Total Score, additional baseline-adjusted analyses were performed using analysis of covariance (ANCOVA), with treatment group as the fixed factor and the corresponding baseline score entered as a covariate. Before performing the ANCOVA analyses, the homogeneity of regression slopes assumption was assessed by testing the interaction between treatment group and the corresponding baseline score. As none of the interaction terms were statistically significant (all *p* > 0.05), the assumption was met.

A *p*-value < 0.05 was considered statistically significant.

## 3. Results

### 3.1. Participants and Baseline Characteristics

A total of 61 patients were assessed for eligibility, of whom 40 met the inclusion criteria and were randomly allocated to the US and SWD groups (*n* = 20 per group). All participants completed the treatment protocol and follow-up assessments, and no losses to follow-up were recorded.

Baseline demographic and clinical characteristics were generally comparable between groups. No statistically significant differences were observed in age, body mass index, symptom duration, sex distribution, CTS severity, MNCSA, grip strength, VAS pain, or DN4 score (all *p* > 0.05). However, baseline Boston Functional Status Score (*p* = 0.024) and Boston Total Score (*p* = 0.026) were significantly higher in the SWD group, whereas the difference in Boston Symptom Severity Score did not reach statistical significance (*p* = 0.056). Baseline demographic and clinical characteristics of the participants are presented in Table 1.

### 3.2. Outcomes

*Primary Outcome:* For the primary outcome, MNCSA, repeated measures ANOVA demonstrated a significant time effect (F = 55.174, *p* < 0.001, η^2^p = 0.592), indicating a reduction in MNCSA over the follow-up period in both groups. However, no significant group × time interaction effect was detected (F = 0.072, *p* = 0.881, partial η^2^ = 0.002), suggesting no statistically significant difference between treatment modalities in terms of MNCSA change over time.

*Secondary Outcomes:* Significant time effects were also observed for all secondary continuous outcomes, including Boston Total Score (η^2^p = 0.729), Boston Symptom Severity (η^2^p = 0.745), Boston Functional Status (η^2^p = 0.605), VAS pain (η^2^p = 0.625), grip strength (η^2^p = 0.607), and continuous DN4 scores (η^2^p = 0.512) (all *p* < 0.001). In each case, group x time interaction effect was non-significant (all *p* > 0.05), indicating that the magnitude of improvement over time did not differ significantly between the US and SWD groups. The post-treatment outcomes are presented in Table 2.

**Figure 2:**
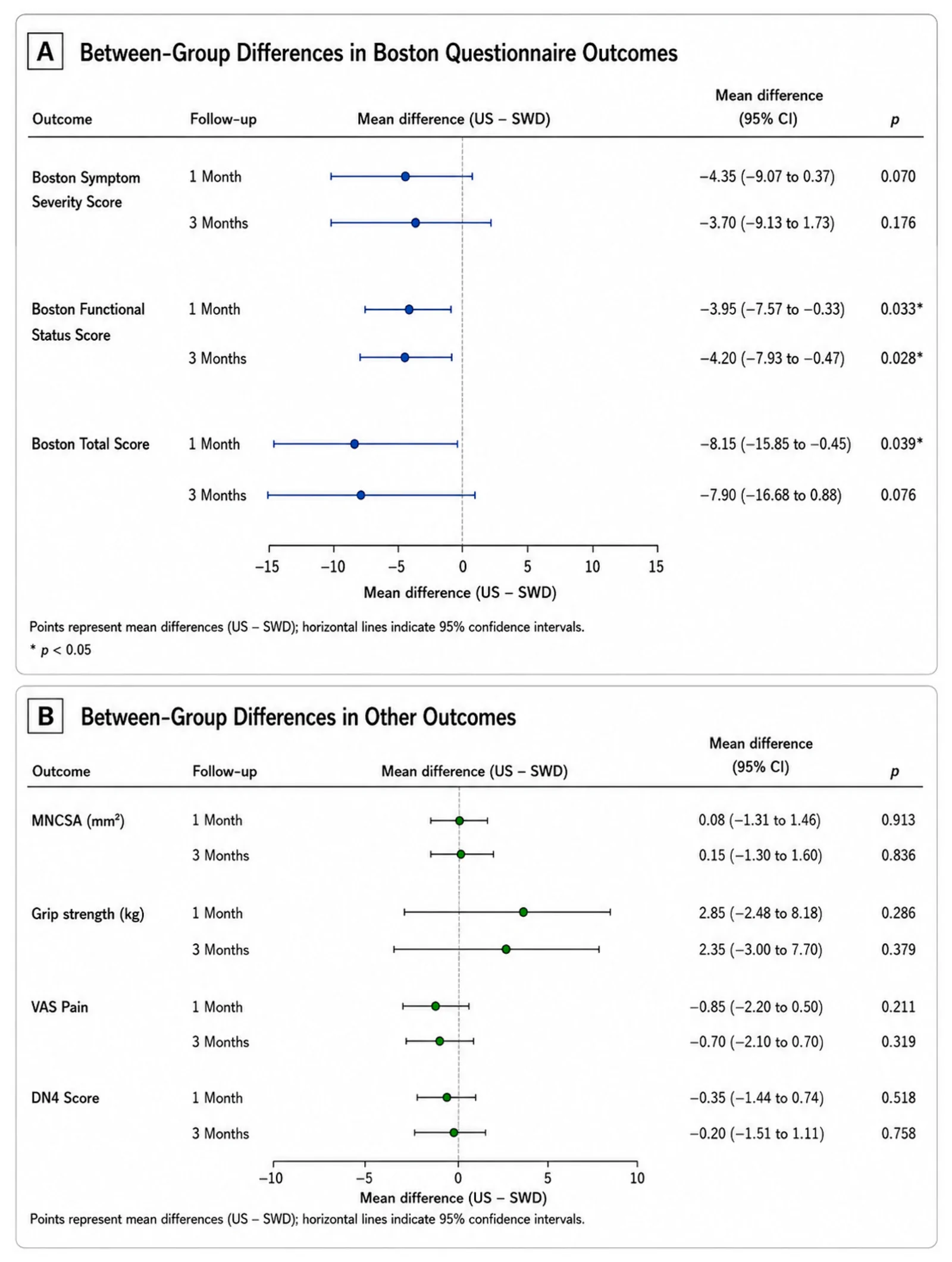
Forest plots showing the unadjusted between-group mean differences with 95% confidence intervals (CIs) at the 1- and 3-month follow-up assessments. (**A**) Boston Questionnaire outcomes. (**B**) Median nerve cross-sectional area (MNCSA), grip strength, Visual Analog Scale (VAS) pain, and Douleur Neuropathique 4 (DN4) scores. Mean differences were calculated as US − SWD. Horizontal lines represent 95% confidence intervals, and the vertical reference line at zero indicates no between-group difference.

Between-group comparisons at the follow-up assessments are presented in Table 3 and graphically illustrated in Figure 2. At the 1-month follow-up, the US group showed significantly lower Boston Functional Status Score (mean difference = −3.95, 95% CI: −7.57 to −0.33; *p* = 0.033) and Boston Total Score (mean difference = −8.15, 95% CI: −15.85 to −0.45; *p* = 0.039) than the SWD group. No significant between-group differences were observed for the remaining outcomes at either the 1- or 3-month follow-up (*p* > 0.05 for all comparisons).

Because baseline differences were observed in the Boston Questionnaire scores, additional baseline-adjusted ANCOVA analyses were performed. After adjustment for the corresponding baseline values, the initially observed between-group differences in Boston Functional Status Score and Boston Total Score were no longer statistically significant, and no significant between-group treatment effects were observed for the Boston Symptom Severity Score, Boston Functional Status Score, or Boston Total Score at either the 1- or 3-month follow-up (all *p* > 0.05) (Table 4).

Consistent with the continuous DN4 analysis, categorical evaluation using the ≥4 cut-off revealed a significant reduction in neuropathic pain prevalence over time in the overall sample (*p* = 0.001). Within-group analyses demonstrated significant reductions in both the US group (*p* = 0.011) and the SWD group (*p* = 0.006). No significant between-group differences were observed at any assessment point (all *p* > 0.05).

## 4. Discussion

In this randomized, assessor-blinded study, we compared the effects of pulsed US and pulsed SWD, administered in addition to splinting and exercise therapy, on clinical outcomes, neuropathic pain, handgrip strength, and MNCSA in patients with electrophysiologically confirmed mild-to-moderate CTS. Significant improvements over time were observed in both clinical and ultrasonographic outcomes within each treatment group; however, no significant group × time interaction was detected, indicating that neither modality demonstrated statistical superiority within the context of the treatment protocol used in this study.

Conservative management of CTS primarily consists of splinting and tendon–nerve gliding exercises, which are widely recommended in the literature [12]. In the present study, both groups received standardized splinting and exercise therapy, and deep-heating modalities were administered as adjunctive interventions. The overall pattern of improvement observed over time is consistent with that reported in previous studies of structured conservative treatment for CTS. Because both treatment groups received identical splinting and tendon–nerve gliding exercises, part of the observed improvement is likely attributable to these established conservative interventions. This approach is broadly consistent with previous randomized controlled trials evaluating physical therapy modalities for CTS, in which wrist splinting was routinely provided as background treatment, while some protocols also incorporated tendon–nerve gliding exercises [6,7,12,13]. Consequently, the present study design cannot isolate the specific contribution of the physical therapy modalities from the effects of background conservative treatment.

One of the notable statistical findings of the present study was the apparent between-group difference observed in the unadjusted BCTQ Functional Status and Total scores at the 1-month follow-up. However, because baseline differences were present between the groups, these outcomes were re-evaluated after adjustment for the corresponding baseline values using ANCOVA. Following this re-evaluation, no statistically significant between-group differences were observed at either the 1- or 3-month follow-up. These findings suggest that baseline differences between the groups may have influenced the interpretation of the post-treatment outcomes, and that adjustment for baseline values allowed a more appropriate evaluation of the follow-up results.

Therapeutic ultrasound has been evaluated in several randomized controlled trials in patients with CTS, with most studies demonstrating improvements in pain, symptom severity, functional status, and selected electrophysiological parameters following treatment [13,22,23]. However, these studies primarily focused on clinical and electrophysiological outcomes, whereas ultrasonographic structural changes were rarely assessed. Armağan et al. reported significant improvements in VAS and BCTQ scores following both continuous and pulsed ultrasound compared with placebo, together with favorable changes in selected nerve conduction parameters. Similarly, the systematic review and meta-analysis by Peris Moya et al. [22] confirmed beneficial effects on distal motor latency and clinical symptom scores, while ElMeligie et al. [23] also demonstrated significant improvements in pain, functional status, and grip strength after both thermal and pulsed ultrasound. In contrast to these previous studies, the present study incorporated ultrasonographic assessment of the MNCSA as a structural outcome. The significant reduction in MNCSA observed alongside clinical improvement suggests that ultrasonographic evaluation may provide complementary information regarding treatment response beyond conventional clinical outcome measures.

Although evidence regarding SWD in CTS is relatively limited compared with US, available randomized controlled trials have generally reported short-term improvements in pain intensity, symptom severity, and functional outcomes following pulsed SWD treatment [6,7]. Similar to most ultrasound studies [6,7,13,22], these trials primarily evaluated symptom-based scales and electrophysiological parameters, whereas ultrasonographic structural outcomes were not routinely included. In the present study, significant within-group improvements were observed in VAS, BCTQ, DN4, grip strength, and MNCSA in the SWD group.

In the present study, MNCSA values decreased significantly over time in both groups. Previous research has suggested that MNCSA may serve not only as a diagnostic parameter but also as a useful marker for monitoring treatment response in CTS [24,25]. Consistent with previous interventional studies, the reduction in MNCSA observed in our study occurred alongside improvements in clinical outcomes [10,24,25]. Cartwright et al. highlighted the value of ultrasonographic assessment of MNCSA as a complementary tool for the diagnosis and follow-up of CTS, supporting its role in evaluating structural changes associated with the disease [15]. Similarly, Lin et al. demonstrated that reductions in MNCSA following ultrasound-guided treatment occurred in parallel with clinical improvement, suggesting that structural and clinical recovery may coexist [10]. The approximately 1 mm^2^ reduction observed in our study, together with the concurrent improvements in pain, functional status, grip strength, and neuropathic pain, is consistent with these findings. Taken together, the available evidence suggests that reductions in MNCSA generally accompany improvements in patient-reported symptoms and functional outcomes following treatment, supporting its role as a complementary imaging marker of recovery rather than a standalone measure of treatment success [26]. Recent studies have also shown that postoperative MNCSA is associated with achieving the minimal clinically important difference in patient-reported outcomes, although this reflects the clinical relevance of MNCSA rather than establishing a minimal clinically important difference for MNCSA itself [27]. To date, no validated minimal clinically important difference or universally accepted threshold has been established for changes in MNCSA. Therefore, changes in MNCSA should be interpreted together with clinical and functional outcomes rather than as an isolated marker of treatment response. With regard to neuropathic pain, the available literature evaluating physical modalities such as US or SWD in CTS remains limited. Most randomized studies have focused primarily on overall pain intensity (e.g., VAS), functional status (e.g., BCTQ), and electrophysiological parameters, rather than neuropathic pain components assessed by dedicated questionnaires [6,7,9,10,14]. Dandinoğlu et al. [28] reported significant reductions in DN4 and LANSS scores following local corticosteroid injection, suggesting that the neuropathic component of CTS may be responsive to therapeutic intervention.

In the present study, both pulsed US and pulsed SWD were associated with significant reductions in DN4 scores over time. Although no between-group difference was detected, the consistent improvement observed across both groups indicates that neuropathic pain symptoms may improve within structured conservative management. These findings add further data to the limited literature addressing neuropathic pain outcomes in CTS.

## 5. Limitations of the Study

The present study has several limitations. First, the trial included two active treatment arms without a control group receiving splinting and exercise alone or a sham intervention. Consequently, the independent therapeutic effects of pulsed US and pulsed SWD cannot be clearly distinguished from the effects of background conservative treatment, placebo responses, or the natural course of CTS. Furthermore, because the study was not designed as a superiority or non-inferiority trial, the absence of significant between-group differences should not be interpreted as evidence of equivalence between the two treatment modalities. Although modest baseline differences were observed in the BCTQ Functional Status and Total scores, additional baseline-adjusted ANCOVA analyses yielded findings consistent with the primary analyses, suggesting that these initial differences did not materially influence the overall interpretation of the results. Electrophysiological parameters were not reassessed after treatment, limiting the ability to explore potential neurophysiological correlates of the observed clinical and ultrasonographic changes. In addition, the follow-up period was limited to three months, precluding conclusions regarding the long-term durability of treatment effects. The study population consisted predominantly of women (approximately 85–90%), reflecting the epidemiology of CTS but potentially limiting the generalizability of the findings to male patients. Although outcome assessors were blinded, participants and treating therapists could not be blinded because of the nature of the interventions, introducing the possibility of performance and placebo effects. Finally, although the sample size was determined based on an a priori power analysis, the calculation was based on a relatively large anticipated effect size. Therefore, the study may have been underpowered to detect smaller, yet clinically meaningful, differences between the two active treatment groups. Future studies incorporating sham or conservative-treatment-only control groups, larger sample sizes, longer follow-up periods, post-treatment electrophysiological assessments, and adequately powered superiority, non-inferiority, or equivalence designs with predefined clinical margins are warranted to better define the modality-specific effects of pulsed US and pulsed SWD in CTS.

## 6. Conclusions

In patients with mild-to-moderate CTS receiving structured conservative treatment, both pulsed US and pulsed SWD were associated with meaningful improvements in symptom severity, functional status, neuropathic pain, grip strength, and median nerve cross-sectional area. No statistically significant differences were observed between the two treatment modalities; however, the present study was not designed to establish therapeutic equivalence. Within the limitations of the study, these findings suggest that both pulsed US and pulsed SWD may be considered as adjunctive treatment options in the conservative management of mild-to-moderate CTS. The parallel improvements observed in clinical and ultrasonographic outcomes support the potential value of ultrasonographic assessment of MNCSA assessment as a complementary tool for monitoring treatment response in addition to its established diagnostic role. Further well-controlled, Future studies incorporating sham or conservative-treatment-only control groups, larger sample sizes, longer follow-up periods, and adequately powered non-inferiority or equivalence designs with prespecified non-inferiority margins are needed to better define the modality-specific effects of these interventions and determine whether pulsed SWD provides clinical outcomes comparable to pulsed US.

## Figures and Tables

**Table 1:** Baseline demographic, clinical, and ultrasonographic characteristics of the study participants.

Variable	US Group (*n* = 20)	SWD Group (*n* = 20)	*p*
Age (years)	52.30 ± 10.43	48.65 ± 10.16	0.269
BMI (kg/m^2^)	32.27 ± 4.83	29.60 ± 4.88	0.090
Symptom duration (years)	2.10 ± 2.18	2.35 ± 2.40	0.731
Female, n (%)	17 (85%)	18 (90%)	0.633 *
Male, n (%)	3 (15%)	2 (10%)	0.633 *
Mild CTS, n (%)	9 (45%)	10 (50%)	0.752 *
Moderate CTS, n (%)	11 (55%)	10 (50%)	0.752 *
MNCSA (mm^2^)	13.00 ± 1.97	12.88 ± 2.20	0.851
Grip strength (kg)	19.55 ± 8.26	17.80 ± 7.37	0.484
Boston Symptom Severity Score	30.40 ± 7.10	34.80 ± 7.02	0.056
Boston Functional Status Score	18.95 ± 5.56	23.20 ± 5.91	0.024
Boston Total Score	49.35 ± 11.81	58.00 ± 11.73	0.026
VAS pain	5.25 ± 2.26	6.15 ± 1.89	0.182
DN4 score	5.25 ± 2.09	5.50 ± 1.63	0.677

Abbreviations: US, pulsed ultrasound; SWD, pulsed shortwave diathermy; CTS, carpal tunnel syndrome; BMI, body mass index; MNCSA, median nerve cross-sectional area; VAS, Visual Analog Scale; DN4, Douleur Neuropathique 4 questionnaire. Data are presented as mean ± standard deviation (SD) for continuous variables and as number (percentage) for categorical variables. Continuous variables were compared using the independent-samples *t*-test. Categorical variables were compared using the Pearson χ^2^ test or Fisher’s exact test, as appropriate. * Fisher’s exact test. Statistical significance was defined as *p* < 0.05.

**Table 2:** Clinical and ultrasonographic outcomes at baseline, 1 month, and 3 months and repeated-measures ANOVA results.

Outcome	US Baseline	US 1 Month	US 3 Months	SWD Baseline	SWD 1 Month	SWD 3 Months	Time Effect F	Time Effect *p*	η^2^p	Group × Time F	Group × Time *p*	η^2^p
MNCSA (mm^2^)	13.00 ± 1.97	12.63 ± 2.03	11.98 ± 2.07	12.88 ± 2.20	12.55 ± 2.29	11.83 ± 2.46	55.174	<0.001 <0.001 <0.001	0.592	0.072	0.881	0.002
Grip strength (kg)	19.55 ± 8.26	22.80 ± 9.27	24.30 ± 9.29	17.80 ± 7.37	19.95 ± 7.24	21.95 ± 7.27	58.640	0.001 0.001 0.001	0.607	0.885	0.397	0.023
Boston Symptom Severity	30.40 ± 7.10	22.30 ± 5.94	20.25 ± 7.69	34.80 ± 7.02	26.65 ± 8.51	23.95 ± 9.19	111.010	<0.001 <0.001 <0.001	0.745	0.140	0.796	0.004
Boston Functional Status	18.95 ± 5.56	15.45 ± 4.69	13.90 ± 4.56	23.20 ± 5.91	19.40 ± 6.44	18.10 ± 6.81	58.283	<0.001 <0.001 <0.001	0.605	0.055	0.867	0.001
Boston Total Score	49.35 ± 11.81	37.75 ± 9.74	34.15 ± 11.57	58.00 ± 11.73	45.90 ± 13.85	42.05 ± 15.51	102.210	<0.001 <0.001 <0.001	0.729	0.056	0.871	0.001
VAS Pain	5.25 ± 2.26	3.50 ± 1.85	2.90 ± 2.07	6.15 ± 1.89	4.35 ± 2.34	3.60 ± 2.30	63.226	<0.001 <0.001 <0.001	0.625	0.107	0.821	0.003
DN4 Score	5.25 ± 2.09	4.15 ± 1.59	3.70 ± 1.89	5.50 ± 1.63	4.50 ± 1.79	3.90 ± 2.17	39.819	<0.001 0.001 <0.001	0.512	0.090	0.848	0.002

Abbreviations: MNCSA, median nerve cross-sectional area; US, pulsed ultrasound; SWD, pulsed shortwave diathermy; VAS, Visual Analog Scale; DN4, Douleur Neuropathique 4 questionnaire; SD, standard deviation. Data are presented as mean ± standard deviation (SD). Time and group × time interaction effects were analyzed using two-way repeated-measures analysis of variance (ANOVA). Partial η^2^ values are reported as measures of effect size. Statistical significance was defined as *p* < 0.05.

**Table 3:** Unadjusted between-group mean differences with 95% confidence intervals for primary and secondary outcomes at the 1- and 3-month follow-up assessments.

Outcome	Follow-Up	US Group (n = 20)	SWD Group (n = 20)	Mean Difference (US − SWD)	95% CI	*p*
MNCSA (mm^2^)	1 Month	12.63 ± 2.03	12.55 ± 2.29	0.08	−1.31 to 1.46	0.913
	3 Months	11.98 ± 2.07	11.83 ± 2.46	0.15	−1.30 to 1.60	0.836
Grip strength (kg)	1 Month	22.80 ± 9.27	19.95 ± 7.24	2.85	−2.48 to 8.18	0.286
	3 Months	24.30 ± 9.29	21.95 ± 7.27	2.35	−3.00 to 7.70	0.379
Boston Symptom Severity Score	1 Month	22.30 ± 5.95	26.65 ± 8.51	−4.35	−9.07 to 0.37	0.070
	3 Months	20.25 ± 7.69	23.95 ± 9.19	−3.70	−9.13 to 1.73	0.176
Boston Functional Status Score	1 Month	15.45 ± 4.68	19.40 ± 6.44	−3.95	−7.57 to −0.33	0.033
	3 Months	13.90 ± 4.56	18.10 ± 6.81	−4.20	−7.93 to −0.47	0.028
Boston Total Score	1 Month	37.75 ± 9.75	45.90 ± 13.86	−8.15	−15.85 to −0.45	0.039
	3 Months	34.15 ± 11.58	42.05 ± 15.51	−7.90	−16.68 to 0.88	0.076
VAS Pain	1 Month	3.50 ± 1.85	4.35 ± 2.35	−0.85	−2.20 to 0.50	0.211
	3 Months	2.90 ± 2.07	3.60 ± 2.30	−0.70	−2.10 to 0.70	0.319
DN4 Score	1 Month	4.15 ± 1.60	4.50 ± 1.79	−0.35	−1.44 to 0.74	0.518
	3 Months	3.70 ± 1.89	3.90 ± 2.17	−0.20	−1.51 to 1.11	0.758

Abbreviations: US, pulsed ultrasound; SWD, pulsed shortwave diathermy; MNCSA, median nerve cross-sectional area; VAS, Visual Analog Scale; DN4, Douleur Neuropathique 4 questionnaire; CI, confidence interval. Data are presented as mean ± standard deviation (SD). Mean differences were calculated as the difference between the US and SWD groups (US − SWD). Between-group comparisons at each follow-up assessment were performed using the independent-samples *t*-test, and the corresponding mean differences with 95% confidence intervals (CIs) are presented. Statistical significance was defined as *p* < 0.05.

**Table 4:** Baseline-adjusted analysis of covariance for Boston Questionnaire outcomes at the 1- and 3-month follow-up assessments.

Outcome	Follow-Up	F	*p*	Partial η^2^
Boston Symptom Severity Score	1 Month	0.166	0.686	0.004
	3 Months	0.003	0.956	<0.001
Boston Functional Status Score	1 Month	0.070	0.793	0.002
	3 Months	0.549	0.463	0.015
Boston Total Score	1 Month	0.089	0.768	0.002
	3 Months	0.026	0.873	0.001

Abbreviations: US, pulsed ultrasound; SWD, pulsed shortwave diathermy; η^2^, partial eta squared. Treatment group (US vs. SWD) was entered as the fixed factor, and the corresponding baseline Boston Questionnaire score was included as a covariate. F values, *p* values, and partial η^2^ are reported. Statistical significance was defined as *p* < 0.05.

## Data Availability

The data presented in this study are available from the corresponding author upon reasonable request. The dataset contains individual participant-level clinical and ultrasonographic data collected in a randomized controlled trial. To protect participant confidentiality and to comply with the approved ethics protocol and informed consent procedures, the complete dataset has not been deposited in a public repository. De-identified data necessary to verify the findings can be made available upon reasonable request.
